# Supplementary material for: Evaluating a sexual violence primary prevention program in Australian secondary schools: A protocol for a pilot cluster randomised controlled trial
Source: PLoS One. 2026 Jul 9;21(7):e0350363. doi: 10.1371/journal.pone.0350363 (PMC13349101; doi:10.1371/journal.pone.0350363)
Supplement: S2 File — (PDF) [file pone.0350363.s002.pdf]

Evaluating a Sexual Violence Primary Prevention program (SEP) in Australian Secondary Schools: A Protocol for a Cluster Randomised Controlled Trial

Administrative information

Title

A parallel, two-arm cluster randomised controlled study of Year 9 students at 12 Australian secondary schools to evaluate the effectiveness of a sexual violence primary prevention program on students' knowledge, attitudes and behavioural intentions.

Registry

Australian and New Zealand Clinical Trials Registry https://www.anzctr.org.au/ (registration pending as of March 31, 2025).

Data set

Primary registry and trial identifying number: ANZCTR number pending
Date of registration in primary registry: TBC
Secondary identifying numbers: NA
Source(s) of monetary or material support: Department of Social Services, Australian Government.
Primary sponsor: La Trobe University
Contact for public queries: Vivienne Moyle- Senior Manager, Research Ethics, Integrity & Biosafety. Address: Level 2, David Myers Building (East), La Trobe University Bundoora Telephone: 03 9479 1443 Email: v.moyle@latrobe.edu.au
Public title: Evaluating a Sexual Violence Primary Prevention program (SEP) in Australian Secondary Schools: A Protocol for a Cluster Randomised Controlled Trial
Scientific title: A parallel, two-arm cluster randomised controlled study of Year 9 students at 12 Australian secondary schools to evaluate the effectiveness of a sexual violence primary prevention program on students' knowledge, attitudes and behavioural intentions.
Countries of recruitment: Australia
Health condition(s) or problem(s) studied: Sexual violence, mental health
Intervention: Active comparator - Secondary school students receiving sexual violence primary prevention program. Control comparator – Waitlist of Secondary School students (receiving usual school curriculum) with no receipt of a sexual violence primary prevention program.
Key inclusion and exclusion criteria: Ages eligible for study: Year 9 secondary school students (approximately 13-15 years old). Sexes eligible for study: both.

Inclusion Criteria: Secondary school students in Year 9, secondary school students attending public schools in northern Tasmania.

Exclusion criteria: Secondary school students outside Year 9, private school students, students whose parents opt-out of their young person's participation in the trial.
Study type: Interventional. Allocation: 1:1. Intervention model: parallel assignment. Masking: Blinded outcomes assessor. Primary purpose: prevention.
Date of first enrolment: June 2025
Target sample size: Clusters n=12; Students n=180
Recruitment status: Recruitment not yet commenced
Primary outcome(s):

- *Knowledge* about sexual violence including gender equality, consent, healthy relationships, and rape myths

Key secondary outcomes:

- *Attitudes* toward gender equality, rape myths, and empathy;
- *Behavioural intentions* associated with consent and bystander intervention.
- *Implementation outcomes* e.g., program reach and retention, acceptability, fidelity, sustainability, and testing realist program theories.

Protocol version

Issue Date: 31 March 2025
Protocol Amendment Number: 01
Author(s): Haylett, F et al.

Funding

The Australian Department of Social Services is funding this study as part of the Partners in Prevention of Sexual Violence project. The project is being run by researchers from La Trobe University and has received funding to evaluate nine sexual violence primary prevention programs across Australia.

Roles and Responsibilities

Protocol Contributors

Dr Freda Haylett, Dr Jacqueline Kuruppu, Professor Leesa Hooker, Dr Jacqui Theobald, Dr Innocent Mwatsiya, Dr Jess Ison, Associate Professor Kirsty Forsdike, Felicity Young - Violet Vines Marshman Centre for Rural Health Research, La Trobe University

Dr Gabriel Caluzzi - Centre for Alcohol Policy Research, La Trobe University

Dr Sarah Vrankovic and Professor Adam Bourne - Australian Research Centre in Sex, Health and Society, La Trobe University

Professor Nicola Henry - Social Equity Research Centre, RMIT University

Dr Kerryn O'Rourke - Charles Darwin University

Dr Xia Li – Statistician, La Trobe University

Author contributions: LH, JI, KF, FY, JT, GC, AB, & NH conceived of the trial and led the trial design, overall analysis plan (with lead support from FH), and the funding application. FH, LH, JI, KF, JT, GC, AB, NH, XL, JK, IM & SV will draft the manuscript. All authors will contribute to refinement of the study protocol and will read and approve the final manuscript.

Sponsor contact information

Trial Sponsor: La Trobe University
Sponsor's Reference**:** 2411977
Contact name: Vivienne Moyle- Senior Manager, Research Ethics, Integrity & Biosafety
Address: Level 2, David Myers Building (East), La Trobe University Bundoora
Telephone: 03 9479 1443
Email: v.moyle@latrobe.edu.au

Sponsor and funder

This study is funded by the Federal Department of Social Services, Australia. The Department of Social Services had no role in the design of this study and will not have any role during its execution, analyses, interpretation of the data, or decision to submit results.

Committees

Principal Investigator and Research Fellow roles

- Design and conduct of the trial
- Preparation of protocol and revisions
- Preparation of data collection materials
- Recruitment of participants with support from Schools and Laurel House
- Organising Trial Management Committee meetings
- Publication of study reports

Trial Management Committee - TBC

- Agreement of final protocol and if necessary, recommending changes to the protocol
- Organising data monitoring committee
- Assist with ethics committee applications
- Responsible for trial master file
- Data verification
- Randomisation

Data Monitoring Committee – Independent, external members TBC

- Biannual meetings to review trial management and progress

- Oversee statistical analysis
- Make recommendations to the Trial Management Committee
- Protect and serve trial participants, to assist and advise the lead, chief (CIs) and associate investigators and therefore protect the legitimacy and integrity of the trial;
- Safeguard the interests of trial participants, assess the safety and efficacy of the intervention during the trial and monitor the overall conduct of the study.

Introduction

Background and rationale

Sexual violence and primary prevention

Sexual violence is a global issue and a significant public health concern. Over 1 in 5 (22%) women and 1 in 16 (6.1%) men in Australia have experienced sexual violence since the age of 15 (ABS, 2023). Research indicates that sexual violence is a gender-based issue arising from gendered inequality and power imbalances (Our Watch et al., 2015; World Health Organization, 2013). It is therefore important that primary prevention efforts "aim to address the gendered drivers of violence against women, including the structures, norms and practices that maintain a gender unequal society" (Our Watch, 2019, p. 13). There are several known risk factors for sexual violence perpetration. These include endorsement of gender inequality, alcohol and other drug use (Gibbs et al., 2020), exposure to family violence (Borowsky et al., 1997), rape myth acceptance and victim-blaming (Lanier, 2001; Maxwell et al., 2003), child maltreatment (Krahé & Berger 2017), poverty, and cultural norms that normalise sexual violence (Heise, 1998; Benson et al., 2003). However, protective factors and conditions can be built to prevent sexual violence, such as strengthening supportive relationships with parents and peers, and reducing social inequities, thereby improving social cohesion (Dills et al., 2019; Coker et al., 2002). Individual-level factors such as empathy, empowerment and interpersonal skills are also important protective mechanisms (Tharp et al., 2013).

There is limited evidence of effective primary prevention interventions, which are intended to prevent sexual violence before it occurs (Our Watch, 2020). Secondary school settings represent a promising area for primary prevention efforts, because interventions can reach participants at a young age, before perpetration may have occurred and at a time in the life course where norms and values are being shaped (De Gue at al., 2014; Porat et al., 2024). The prevention of gender-based violence and sexual violence was a focus of the Australian Government's *National Plan to Reduce Violence against Women and Their Children 2010-2022* (Council of Australian Governments, 2011). Preventing violence continues as a priority in the Australian Government's current policy framework *The National Plan to End Violence Against Women and Children 2022-2032,* which recognises school-based consent and respectful relationships education programs as a strategy to reduce sexual violence and recommends embedding prevention approaches in schools (Commonwealth of Australia, 2022).

Whilst some interventions have demonstrated success in increasing knowledge, awareness and skills relating to sexual violence, there are limited examples of interventions that have changed perpetrator behaviours (Porat et al., 2024; De Gue et al., 2014). By way of example, the US-based *Safe Dates* intervention aimed to reduce dating violence among secondary school students. Researchers found that students who received the intervention 'reported significantly less physical, serious physical, and sexual dating violence perpetration and victimisation 4 years after the program' (Foshee et al., 2004, p. 619). Another school-based intervention implemented in the United States, *Shifting Boundaries,* targeted students in grades 6 and 7 with an innovative dual strategy. It delivered an intervention in the classroom alongside schoolwide measures such as revising school protocols, introducing a temporary school-based restraining orders, and displaying posters around the school. For the students who received both the classroom and schoolwide interventions, or the schoolwide intervention alone, behaviours improved post-intervention (Taylor et al., 2013). A bystander engagement strategy called *Green Dot* was employed by another intervention, in secondary schools with the aim of reducing sexual violence. This was evaluated and shown to produce lower rates of violence victimisation and perpetration (Coker et al., 2015).

These interventions demonstrate some positive effects on knowledge, attitudes, skills and behaviour; however, most are not sexual violence-specific, and all were developed for American education settings. Therefore, applicability to Australian schools may be a challenge (Hooker et al., 2020). In the Australian context, no peer-reviewed studies have been conducted on primary prevention interventions to address sexual violence (Hooker et al., 2020).

Rationale for proposed study

Given the prevalence of sexual violence and its harmful impacts, there is a critical need for effective primary prevention interventions targeting school settings in Australia. Schools are sites where young adolescents are socialised, and these early socialisation experiences shape behavioural norms and can form developmental pathways that enable the perpetration of sexual violence (Nagayama & Barongan, 1997).

To address this need, the Partners in Prevention of Sexual Violence project received funding from the Australian Federal Department of Social Services to adapt and evaluate an existing program titled the 'Schools Education Program' (SEP) delivered by sexual assault support service Laurel House to Tasmanian secondary schools. The intervention will be evaluated using a cluster randomised controlled trial (cRCT) to assess the effects of SEP on changes to Year 9 students' knowledge, attitudes and behavioural intentions.

Choice of comparators

To better understand the role of consent and healthy relationships education in the prevention of sexual violence, we will measure outcomes across 12 schools in Tasmania, Australia. Of the 12 schools, six schools will be randomly assigned to the intervention group and six schools to the waitlist control group. The control group will not receive the prevention program during the trial, but for ethical reasons will receive the intervention in 2026 after the study has concluded.

Objectives

The objectives of the trial are to:

1) Primarily determine the effectiveness of the intervention in improving students' knowledge about gender equality and sexual violence including consent, healthy relationships, and rape myths
2) Secondly, determine student attitudes and behavioural intentions regarding gender equality and sexual violence
3) Conduct a process evaluation to assess the program reach, retention, fidelity, acceptability, and sustainability of the intervention
4) Qualitatively assess the contextual factors and underlying mechanisms that contributed to the primary and secondary outcomes using a realist approach.

Trial design

A parallel two-arm cluster RCT will be conducted among Year 9 students aged 13-15 years old in 12 secondary schools across Tasmania, Australia from 2025 to 2026. With assistance from a statistician, and using minimisation and stratified randomisation techniques, schools will be 1:1 allocated to either a sexual violence primary prevention intervention (SEP), or to a waitlist control group. The level of randomisation will occur at the school level (cluster), rather than the individual level, because of the high risk of contamination in schools where students are routinely interacting. A comprehensive process evaluation and realist evaluation will be conducted alongside the cluster RCT.

Methods

Participants, interventions and outcomes

Study setting

SEP is an educational intervention intended for Year 9 students in secondary school.

Eligibility criteria

The inclusion criteria are:

1) students attending public secondary schools in Northern Tasmania, Australia

2) students in Year 9-aged 13-15 in these schools, unless their parent/caregiver opt-out of their participation in the trial.

The exclusion criteria are:

1) students attending private schools,

3) students whose parents opt-out of their participation in the trial.

4) students outside Year 9

SEP will be delivered in schools that are both socio-economically and socio-educationally disadvantaged, and are in regional, rural and remote areas of northern Tasmania.

Intervention

The SEP educational intervention aims to educate students on healthy sexual relationships and prevent sexual violence by enhancing student knowledge, attitudes and behavioural intentions.

Year 9 students in the intervention arm will receive three x 90-minute sessions of SEP facilitated by Laurel House, in partnership with the Tasmanian Women's Legal Service.

Session One

In the first session, facilitators will use small-group activities and open discussion to explore the following topics: 1) healthy relationships, including communication, boundaries, mutual respect and consent, 2) defining sexual violence, common misconceptions about sexual violence, and recognising its impact on victim-survivors, 3) gender equality and harmful stereotypes, law changes over time that have affected women's roles, gendered societal expectations, and how these influence perceptions of sexual violence.

Session Two

This session delves into legal aspects of consent and emerging forms of sexual violence. With expert insights from a women's legal service lawyer, the session will explore: 1) legal definitions of consent, 2) image-based abuse, 3) non-fatal strangulation (sexual choking), stealthing and the law, 4) and guidance on accessing legal support and counselling.

Session Three

The final session for students will focus on navigating consent, digital safety, and active bystander strategies. Using discussion and small-group scenarios, facilitators will focus on 1) building skills for clear, respectful dialogue about boundaries and mutual agreement in relationships, 2) how pornography shapes perceptions of sex, relationships, and unrealistic expectations. 3) reinforcing the serious legal consequences of accessing, sharing, or producing Child Sexual Abuse Material, and its lifelong harms to victim-survivors. 4) early warning signs and strategies to address harmful sexual behaviours, and 5) practical bystander strategies to safely challenge harmful behaviours, support potential victims, and disrupt unsafe situations.

In addition to educational sessions, there will be 'School Student Champions' recruited at each school to help accelerate the diffusion of messages in a population, an evidence-based strategy employed in similar interventions (Cook-Craig et al., 2014; Valente & Davis, 1999). Additionally, members of the school staff and parents/caregivers will be invited to attend a 2-hour educational session on consent and healthy relationships. Year 9 students in the control arm will not receive the program and will instead receive their usual school curriculum.

SEP is an age-appropriate intervention guided by the Australian government's *Commonwealth Consent Policy Framework: Promoting healthy sexual relationships and*

consent among young people (Department of Social Services, 2023), as well as the gendered drivers of violence outlined in *Change the Story* (Our Watch et al., 2015) and the societal-level and practical barriers to primary prevention of sexual violence set out in the *Theory of Change* (Hooker et al., 2021). The Commonwealth framework was developed as a practical, evidence-based guide targeted at young people, with the aim of preventing sexual violence. SEP uses a variety of teaching methods and creative props to educate young people on the skills needed for healthy sexual relationships, including communicating consent; balancing the pleasurable aspects of sexual activity with the harms of sexual violence; and the role of gendered stereotypes in driving sexual violence. The intervention content is inclusive of a diverse range of sexual experiences, gender identities, and sexual orientations. See Figure 1 for the SEP logic model.

**Inputs**	**Activities**	**Short-term outcomes**	**Medium-term outcomes**	**Long-term outcomes**
Program materials<br><br>Full-time educators, Part-time practice champion<br><br>Regular internal and external supervision<br><br>Partnership with Women's Legal Service Tasmania<br><br>Evidence-based practice<br><br>Laurel House expertise and readiness<br><br>School buy-in and readiness<br><br>Crisis and counselling support<br><br>In-kind funding<br><br>Funding from Department of Social Services (DSS)	Resource development<br><br>Co-design with schools<br><br>Program adaptation<br><br>Educational session with school staff<br><br>Educational session with parents/carers<br><br>Educational sessions with students<br><br>School Student Champions<br><br>Management of disclosures by students, parents or school staff<br><br>Post-program reflection and revision<br><br>Managing key stakeholder relationships	Consistent student attendance and engagement at workshops<br><br>Teachers are upskilled and confident to take messages forward beyond program<br><br>Attendance and engagement of teachers and parents/carers at educational sessions<br><br>Increased knowledge and awareness among students about consent and the law; porn literacy; the gendered drivers of sexual violence; and how to access support services.	Adoption of respectful and consensual behaviours in relationships<br><br>Positive attitude shifts in relation to myths about sexual violence and gender stereotypes<br><br>Increased disclosures by victim-survivors<br><br>Improved student skills in relation to bystander techniques and understanding consent<br><br>Increased referrals to specialist services	Promote change to the school's socioecology on an individual, relational, and community level to address the drivers of sexual violence<br><br>Better informed and responsive parents and carers<br><br>Advance the evidence-base for primary prevention interventions in school settings

Figure 1: Schools Education Program logic model

Outcomes

The primary outcome for this trial is:

- Improvements in students' **knowledge** about gender equality and sexual violence including consent, healthy relationships, and rape myths

The secondary outcomes are:

- Improvements in students' **attitudes** toward gender equality, rape myths, and empathy
- Improvements in students' **behavioural intentions** associated with consent and bystander intervention
- **Implementation outcomes**- fidelity, reach and retention, acceptability, sustainability.

The primary and secondary outcomes are measured using self-reported changes to students' knowledge from a 22-item scale developed for this study. This scale is theoretically underpinned by existing validated outcomes measures (detailed below) from studies on gender-based violence, general violence, or sexual violence. The items have been modified to simplify the language and/or to ensure the questions are appropriate for Year 9 students. Changes in mean scores will be analysed from baseline to immediate post intervention, then six months post and across trial arm.

Outcome measures

Student Knowledge

The survey instrument comprises six items assessing participants' knowledge related to relationships and empathy. Respondents indicate their level of agreement with statements such as "I know what a healthy relationship looks like" using a 5-point Likert scale (1 = Strongly disagree to 5 = Strongly agree).

Student Attitudes

Attitudes will be assessed using six items measuring gendered beliefs, including statements such as "Boys are better leaders than girls" and "Girls often say 'no' to sex when they really mean 'yes'." Responses will be recorded on a 5-point Likert scale ranging from 1 (Strongly disagree) to 5 (Strongly agree).

Student Behavioural Intentions

The survey includes four items assessing behavioural intentions related to consent and bystander intervention. Participants are presented with statements beginning with "I would…" and are asked to rate their responses on a 4-point Likert scale (ranging from 0 to 3), indicating their anticipated frequency of engagement in each behaviour.

Implementation outcomes

In line with Medical Research Council Guidelines for complex interventions, a comprehensive process evaluation will take place, using mixed methods approaches to examine intervention reach, retention, fidelity, acceptability, and sustainability.

Reach, Retention and Fidelity-facilitators delivering the intervention will record student attendance and other challenges or patterns of implementation using fidelity sheets. These sheets will be completed by facilitators after each student session.

Acceptability and sustainability-assessed via interviews with students, facilitators and other key stakeholders.

Participant timeline

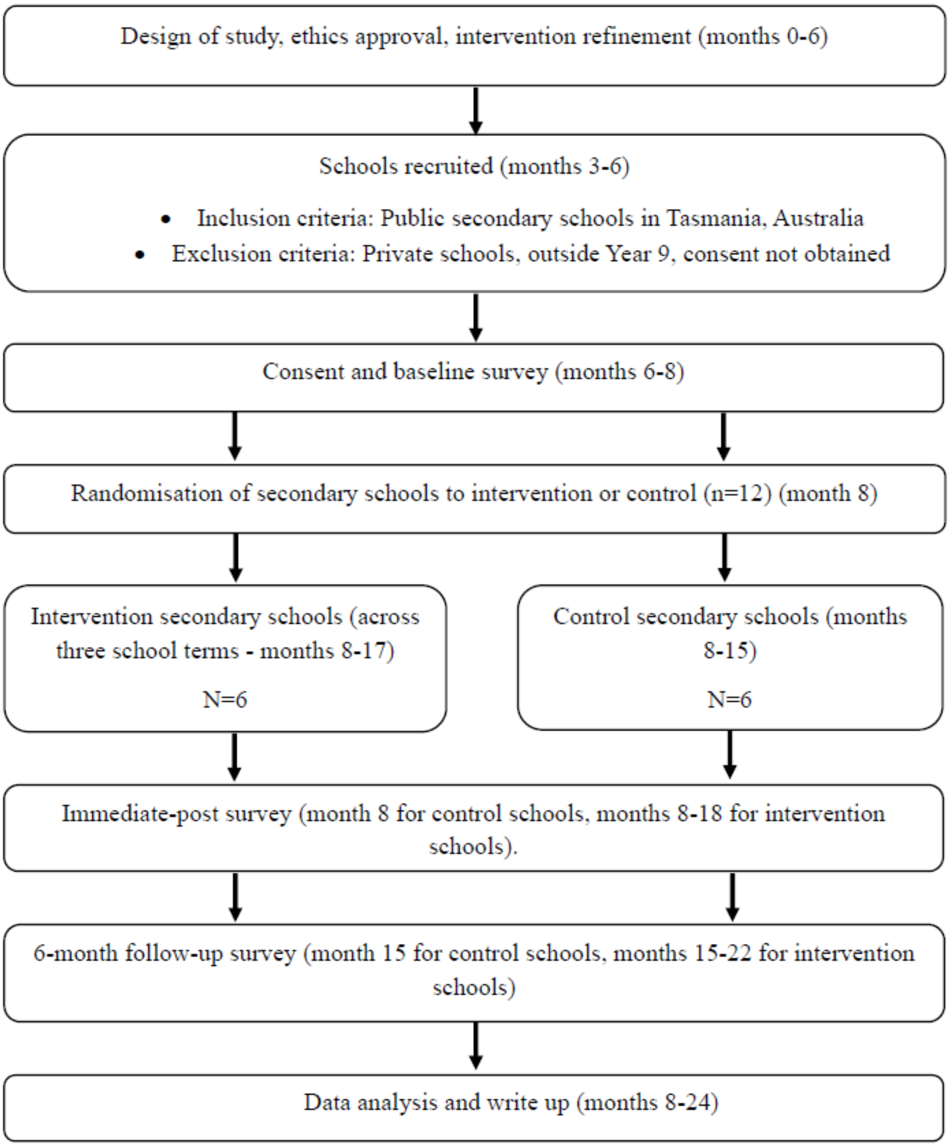

Figure 2: Study flow chart

All students will complete a baseline survey before randomisation, immediately-post intervention, and again at 6-month follow-up. A baseline survey will be completed by students before the commencement of the program in schools. An immediate-post survey and later a 6-month follow-up survey will also be administered. Students will complete the surveys in their own time online through REDCap, and each survey will be open for one

week. Students whose parents/caregivers have consented to their participation will be provided with a Participant Information and Consent Form (PICF) beforehand explaining the purpose of the research and its attendant risks and benefits, as well as the secure and confidential handling of their data. Students who consent to participate will receive a baseline survey which will collect data on demographic and potential confounder variables, as well as hypothesised outcome variables. Immediate-post surveys will contain the same hypothesised outcome variables. Six-month follow-up surveys will contain an additional item intended to collect self-reported data on which program sessions the students found most impactful.

Sample size

A parallel, two-group cluster-randomized design will be used to test whether the Group 1 (Intervention) knowledge average level ($\mu1$) is greater than the Group 2 (control) average level ($\mu2$) (H0: $\delta \leq 0$ versus H1: $\delta > 0$, $\delta = \mu1 - \mu2$). Based on similar school-based dating violence prevention interventions in the USA (Lee & Wong, 2020), the assumed effect size for the primary outcome (knowledge) is 0.566, corresponding to a standardised mean difference (SMD) of 0.566. To detect this mean difference with 80% power, 6 clusters will be assigned to Group 1 and 6 to Group 2. The required average sample size per cluster is 12 for both groups, yielding a total of 72 subjects per group. Sample size calculations were performed using PASS 2024 (version 24.0.1).

The comparison will be conducted using a one-sided t-test, with degrees of freedom based on the total number of subjects and a Type I error (α) of 0.025. Assumptions include a common subject-to-subject standard deviation of 1, an intra-cluster correlation coefficient of 0.026, and a coefficient of variation for cluster sizes of 0.70926. To account for an estimated student dropout rate of approximately 20%, the required average sample size per cluster is increased to 15, resulting in a total of 180 students.

Recruitment

The secondary schools will be recruited by Laurel House, leveraging their existing networks to directly recruit eligible schools. Many schools have shown interest in offering the program (n=12 with student numbers approx. 900). The research team will gain consent from parents/caregivers prior to their young persons' participation in the trial.

Assignment of intervention

Allocation-Sequence generation, concealment mechanism, implementation and blinding

Schools (clusters) will be randomised using a remote system set up by the LTU Statistics Platform. The allocation sequence will be stored on a secure server and concealed from all personnel involved in the trial. It will be created, using computer-generated random numbers, by a statistician with no link to, or contact with, any of the participating schools. Randomisation will be stratified by the following five variables:

1. Year 9 student population (size)
2. Region of Tasmania
3. Index of Relative Socio-Economic Disadvantage, by suburb and locality (IRSD)
4. Indigenous enrolment, percentage
5. Index of Community, Socio-Educational Advantage (ICSEA)

Once all schools have been confirmed and baseline forms completed, LH will contact the Statistics Platform requesting the randomised assignment of all schools. These assignments will then be communicated to the schools by FH. Prior to baseline form completion all trial team members, schools, and students will be blinded; after this point, all will be unblinded.

Data collection, management and analysis

Data collection methods and participant retention

Survey

As there are no survey instruments capturing the objectives and characteristics of this study, the research team developed a survey tool for this trial. Survey instrument development was theoretically informed by the following existing validated outcomes measures: self-restraint-Weinberger Adjustment Inventory and the Basic Empathy Scale (BES) (Weinberger & Schwartz, 1990); Attitude Toward Women Scale for Adolescents (AWSA) (Galambos et al., 1985); Couple Violence Scale (ACVS) (Foshee et al., 1992); Dating Self-Protection Against Rape Scale (DSPARS) (Moore & Waterman, 1999); VicHealth 2006 Community Attitudes to VAW Survey (Taylor & Mouzos, 2006); Bystander Attitude Scale-Revised (BAS-R) (McMahon et al., 2014).

The survey items address student knowledge, attitudes and behavioural intention. In designing the survey items, we have been mindful of the cognitive and developmental age of Year 9 students, as well as Tasmanian literacy rates, in which half of Tasmanian 15-year-olds fall short of Australia's proficient reading standard (Thomson et al., 2019). Therefore, the items consist of straightforward and familiar language.

Data collected from student surveys at baseline, immediate post- and at 6-month follow-up will be complemented with data collected from interviews, focus groups and fidelity sheets. Interviews will be conducted with up to 20 students across the six intervention schools and with up to five external stakeholders, such as Senior Social Workers and Regional Safeguarding Leads. Focus groups will be conducted with up to eight program facilitators from Laurel House and Women's Legal Services Tasmania, and with up to 16 school staff members from a cross-section of participating schools.

Interview

The interview schedule for students assesses their experience of the program, the impacts the program has had on them, including changes in knowledge and understanding, changes they would make to the program, and the value they perceive in the program for others their age. The interview schedule for external stakeholders focuses on their views of region-specific needs, of the program and Laurel House's position as a program facilitator. Focus groups with

Laurel House facilitators explore implementation of the program, including experiences of facilitating the program, and the co-design process with schools. This data will be collected in conjunction with fidelity sheets filled out by facilitators which will be used to track student attendance and engagement in the program. The focus group schedule for school staff covers perceptions of the value of the program, experience of the co-design process, the response to the program from parents and students and changes being made to school policies or practices because of the intervention.

Retention

Participant retention will be promoted throughout the study using reminders and vouchers. Students will be sent up to three automated reminders per survey via REDCap email reminders asking them to complete follow-up surveys. Students who participate in the follow-up interview will be offered a $30 gift card to acknowledge their time. The structured school environment in which the program and follow-up will take place will also serve as a mechanism to keep participants engaged throughout the study.

If participants express a desire to discontinue or deviate from intervention protocols, the following data will be collected: reason for discontinuation or deviation; the type of deviation; when the deviation occurred; engagement in any alternative programs of a similar nature and the details of these programs; changes in knowledge, attitudes and behaviour intentions; and buy-in and acceptability of the program. Participants will be encouraged to continue with surveys to assess the impact of deviation or discontinuation, though they will not be obligated to provide this data.

Data management

Only personal data necessary to the outcomes of the overall study will be collected. To ensure confidentiality of participant identities, all data will be securely stored on the La Trobe Research DataSpace (RDS). Identifiable data such as interview, focus group and fidelity sheets will also be held on the project's RDS and labelled with a code that does not link to participant identities. Nonidentifiable survey data will be held on REDCap and the RDS. The REDCap database does not link participant email addresses to their survey responses. Any data that is exported to the statistical analysis platform SPSS will be done in a way that ensures data is de-identified and has user privileges restricted to avoid the improper use of sensitive data. The RDS is accessed via password-protected desktops. Files containing sensitive information will be held in a restricted access folder on the RDS. At the end of the study, LTU researchers will retain materials for seven years after participants have turned 18, in line with the sponsor's archiving policies for health data. After this period, all trial-related items will be destroyed by LTU researchers. The Department of Social Services will separately retain quantitative data from the study indefinitely on their secure database. A Confidentiality Agreement will be drafted with the transcription service to protect sensitive information from being disclosed to unauthorised persons and to ensure researchers and transcribers comply with privacy laws and regulations.

The Data Management Committee will manage and monitor data, assessing safety and quality (See proposed ToR-Appendix O1).

Statistical methods-analysis and missing data

The study results will be reported using the CONSORT guidelines for cluster RCTs. The proportion of students completing baseline, immediate-post and six-month follow-up surveys will be reported. A descriptive analysis will be used to summarise and describe the baseline characteristics (categorical or continuous variables) of the student participants, such as age, gender, sexual orientation, Indigeneity, country of birth, language spoken at home, and autism, ADHD or learning disabilities. For the quantitative outcome measures, a comparison of the intervention and control will be undertaken using SPSS or STATA to determine whether the Group 1 (treatment) mean ($\mu 1$) is greater than the Group 2 (control) mean ($\mu 2$) (H0: $\delta \leq 0$ versus H1: $\delta > 0$, $\delta = \mu 1 - \mu 2$) on the primary outcome of knowledge. This will be repeated for secondary outcomes e.g. attitudes and behavioural intentions. Mean differences on outcomes will be assessed across trial arm and between baseline and post, baseline and follow up and post and follow up. The comparison will be made using a one-sided t-test (alpha 0.025) with the degrees of freedom based on the total number of subjects. Estimates for differences between intervention and control (odds ratios and least squares mean differences) will be adjusted for clustering (students nested within schools) and presented alongside 95% CIs. Intention to treat analysis will be completed and any missing data will be reported and managed using listwise deletion.

Process evaluation

Intervention reach and retention will be assessed by tracking the number of students engaged in the program throughout delivery (n= and proportions reported). Tracking of program participant engagement will occur through fidelity sheets filled out at the end of each student workshop. Fidelity will be measured by facilitators recording deviations from planned content delivery through fidelity sheets. Acceptability and sustainability will be assessed through interviews at the end of the intervention with external stakeholders and program facilitators, paying particular attention to whole-of-school buy-in, resourcing, stakeholder engagement and adaptability.

Qualitative analysis

All interviews and focus group data collected during the process and outcome evaluation will be recorded using a digital device or using the record feature on Teams/Zoom and transcribed by a professional transcription service. Informed by the theory-driven approach of realist evaluation, this study aims to answer *what works*, *for whom*, *under what circumstances* and *how.* We aim to do this by examining how intervention outcomes are determined by mechanisms that are set off in specific contexts, otherwise known as “context-mechanism-outcome configurations” (Pawson & Tilley, 1997; Pawson et al., 2005). Data on context and mechanisms will be collected using interviews with students and external stakeholders and focus groups with program facilitators and school staff. The data will then be analysed thematically, facilitated by NVivo 14, and the realist program theories will be tested and refined. This process of refinement will be iterative, occurring throughout the study as emerging data are collected and analysed.

CMO 1: Respect		
**Context**	**Mechanism**	**Outcome**
Culture of respect within classroom where students give and receive respect	Resource: Classroom teacher demonstrates high regard for their students<br><br>Reasoning: Students feel respected and emotionally safe. Students decide to engage positively with the Laurel House curriculum	Students increase their knowledge and skills, leading to a reduction in harmful sexual behaviours

CMO 2: Expert facilitation

Context	Mechanism	Outcome
Lessons are led by expert facilitators	Resource: The expertise and experience of Laurel House facilitators enables them to confidently and sensitively deliver the lessons<br><br>Reasoning: Students notice the facilitator is highly skilled. They feel trusting and safe	Students build more knowledge about consent and healthy relationships

Monitoring

Data monitoring and interim analysis

A Data Monitoring Committee (DMC) will be established comprising of four independent (from the sponsor) researchers including a chair (TBC). Participants will be external to LTU with at least one international member. The DMC will:

- protect and serve trial participants, to assist and advise the lead, chief (CIs) and associate investigators and therefore protect the legitimacy and integrity of the trial;
- safeguard the interests of trial participants, assess the safety and efficacy of the intervention during the trial and monitor the overall conduct of the study.

Each month of the trial, a member of staff at each enrolled school will report any adverse event arising from the intervention using a REDCap online form.Any adverse events that might plausibly be caused by the intervention will be immediately reported to the governing Human Research Ethics Committee, the Trial Management Committee (TMC) and the Data Monitoring Committee. An adverse event may include but is not limited to 1) a non-life

threating physical injury that requires medical or psychological care, and 2) death, whether by suicide or murder.

Harms and auditing

See SAE reporting template -Appendix O2. Any adverse events and or unintended effects of the trial will be monitored by the DMC and TMC and decisions made to terminate the trial if necessary.

As this is not a clinical trial, formal auditing processes by the sponsor will not occur. See DMC detail on assessment of project conduct, safety and adverse events.

Ethics

This study was approved by the Department for Education, Children and Young People, Tasmania (ref: 2025-06) on March 18, 2025. All study participants will be required to provide informed consent prior to participating in data collection.

Further ethics approval will be obtained from La Trobe University Human Research Ethics Committee, which is compliant with the National Statement on Ethical Conduct in Human Research. The TMC will guide the research and provide advice and support throughout the study. All members of the research team have Victorian issued Working with Children Checks (WWCC), with FH and JT possessing a Tasmanian issued Working with Vulnerable People Check.

Protocol amendments

If there are substantive modifications to the trial protocol which may impact the conduct of the study, such as sample sizes, eligibility criteria, potential benefits or risks to participants, or changes to the study design and outcomes, a formal amendment to the protocol will be required. The amendment will be communicated to ANZCTR, agreed upon by the Trial Management Committee, and approved by the La Trobe University Human Research Ethics Committee prior to the amendment being implemented.

Consent

At the commencement of the trial, a senior staff member at each of the 12 participating schools will send an email out to parents and caregivers of Year 9 students informing them of the planned trial and including a PICF embedded in a REDCap e-Consent form. This will enable the parent/caregiver to 1) consent to their young person's participation in the survey and/or the interview and 2) write their young person's student email address in the available field. A researcher will then distribute PICFs to students whose parent/caregiver has approved of their participation. If a parent/caregiver approves of a student's participation, but the student themselves declines to participate, they will not be enrolled in the trial.

The other participant groups – external stakeholders, school staff and program facilitators will be sent a recruitment script and PICF by a researcher inviting them to participate in the study.

Confidentiality

Survey data collected on REDCap will be securely stored within the REDCap database which does not link participant email addresses to their survey responses. Any data that is exported to a secure statistical analysis platform will be done in a way that ensures data is de-identified and user privileges are restricted to members of the research team, to avoid the improper use of sensitive data. Survey data will be checked by a member of the research team for accidental inclusion of identifiable data, and if found this will be deleted.

All interview and focus group audio recordings and transcripts will be securely held in a La Trobe research drive (RDS) and only accessible to research team members. Audio recordings will be professionally transcribed. Upon return, all transcripts will be de-identified, such as the removal of any names or other associated identifiers.

Declaration of interests

This project is being funded by the Australian Department of Social Services. The funder had no role in study design and will have no role in the collection, management, analysis or interpretation of data. The funder will have no role in writing manuscripts based on study findings or in the decision to submit findings for publication.

The authors declare that they have no competing interests.

Access to data

All CIs and members of the research team listed on the LTU HREC application will have access to the data, except for the Laurel House Practice Champion, Nita Joy.

Dissemination policy

Trial results will be communicated to participants, relevant stakeholders and the public through a final report and publications which will be published on the Partners in Prevention of Sexual Violence website. Laurel House will also disseminate the final report to their professional networks. The findings will be further disseminated through academic and practice conference presentations, the abstracts of which will be posted on Partners in Prevention of Sexual Violence website.

Authorship and Reproducible Research

A detailed publication plan has been developed for the Partners in Prevention project and criteria for authorship negotiated as per ICMJE guidelines. No plans are in place to share the data with the public.

This study is sponsored by La Trobe University and has been approved by the Human Research Ethics Committees (XXXXXX) and the Tasmanian Department for Education, Children and Young People (ref: 2025-06). This trial will be prospectively registered with ANZCTR (identifier: XXXX) on [insert date of registration]. The trial will follow CONSORT guidelines for cluster RCTs, and this protocol follows Standard Protocol Items: Recommendations for Interventional Trials (SPIRIT) guidelines (Chan et al., 2013).

References

Benson, M. L., Fox, G. L., DeMaris, A., & Van Wyk, J. (2003). Neighborhood disadvantage, individual economic distress and violence against women in intimate relationships. *Journal of quantitative criminology*, *19*, 207-235.

Borowsky L. M., Hogan M., Ireland M. (1997). Adolescent sexual aggression: Risk and protective factors. *Pediatrics*, 100, 1–8.

Chan, A. W., Tetzlaff, J. M., Altman, D. G., Laupacis, A., Gøtzsche, P. C., Krleža-Jerić, K., Hróbjartsson, A., Mann, H., Dickersin, K., Berlin, J. A., Doré, C. J., Parulekar, W. R., Summerskill, W. S., Groves, T., Schulz, K. F., Sox, H. C., Rockhold, F. W., Rennie, D., & Moher, D. (2013). SPIRIT 2013 statement: defining standard protocol items for clinical trials. *Ann Intern Med,* 158(3), 200-207. https://doi.org/10.7326/0003-4819-158-3-201302050-00583

Coker, A. L., Bush, H. M., Brancato, C. J., Clear, E. R., & Recktenwald, E. A. (2019). Bystander Program Effectiveness to Reduce Violence Acceptance: RCT in High Schools. *Journal of family violence*, 34(3), 153–164.

Coker, A. L., Fisher, B. S., Bush, H. M., Swan, S. C., Williams, C. M., Clear, E. R., & DeGue, S. (2015). Evaluation of the Green Dot bystander intervention to reduce interpersonal violence among college students across three campuses. *Violence against women*, *21*(12), 1507-1527.

Coker, A. L., Smith, P. H., Thompson, M. P., McKeown, R. E., Bethea, L., & Davis, K. E. (2002). Social support protects against the negative effects of partner violence on mental health. *Journal of women's health & gender-based medicine*, *11*(5), 465-476.

Cook-Craig, P. G., Coker, A. L., Clear, E. R., Garcia, L. S., Bush, H. M., Brancato, C. J., Williams, C. M., & Fisher, B. S. (2014). Challenge and Opportunity in Evaluating a Diffusion-Based Active Bystanding Prevention Program: Green Dot in High Schools. *Violence Against Women*, *20*(10), 1179-1202.

Davidov, D. M., Hill, K., Bush, H. M., & Coker, A. L. (2020). The green light for Green Dot: A qualitative study of factors influencing adoption of an efficacious violence prevention program in high school settings. *Violence against women*, *26*(12-13), 1701-1726.

DeGue, S., Valle, L. A., Holt, M. K., Massetti, G. M., Matjasko, J. L., & Tharp, A. T. (2014). A systematic review of primary prevention strategies for sexual violence perpetration. *Aggression and violent behavior*, *19*(4), 346-362.

Department of Social Services (2022). *National Plan to End Violence against Women and Children 2022-2032*. Commonwealth of Australia.

Department of Social Services. (2023). *The Commonwealth consent policy framework,* Commonwealth of Australia.

Foshee V, Fothergill K, Stuart J. (1992). Results from the Teenage Dating Abuse Study conducted in Githens Middle School and Southern High Schools. Technical Report. Chapel Hill, NC: University of North Carolina, (Unpublished).

Foshee, V. A., Bauman, K. E., Ennett, S. T., Linder, G. F., Benefield, T., & Suchindran, C. (2004). Assessing the long-term effects of the Safe Dates program and a booster in preventing and reducing adolescent dating violence victimization and perpetration. *American journal of public health*, *94*(4), 619-624.

Galambos NL, Petersen AC, Richards M, Gitelson IB. (1985). The Attitudes Towards Women Scale for Adolescents (AWSA): a study of reliability and validity. *Sex Roles,* 13(5/6):343-356

Gibbs, A., Dunkle, K., Ramsoomar, L., Willan, S., Jama Shai, N., Chatterji, S., Naved, R., & Jewkes, R. (2020). New learnings on drivers of men's physical and/or sexual violence against their female partners, and women's experiences of this, and the implications for prevention interventions. Global Health Action, 13(1), 1739845.

Heise, L. L. (1998). Violence against women: An integrated, ecological framework. Violence Against Women, 4(3), 262–290.

Hooker, L., Ison, J., Henry, N., Fisher, C., Forsdike, K., Young, F., Korsmeyer, H., O'Sullivan, G., & Taft, A. (2021). Primary Prevention of Sexual Violence and Harassment Against Women and Girls: Combining Evidence and Practice Knowledge - Final Report and Theory of Change. La Trobe University.

Hooker, L., Ison, J., O'Sullivan, G., Fisher, C., Henry, N., Forsdike, K., Young, F., & Taft, A. (2020). Primary prevention of sexual violence and harassment against women and girls: Combining evidence and practice knowledge - Research report. Australian Government Department of Social Services.

Krahé, B., & Berger, A. (2017). Gendered pathways from child sexual abuse to sexual aggression victimization and perpetration in adolescence and young adulthood. *Child Abuse & Neglect*, *63*, 261-272.

Lanier C. A. (2001). Rape myth accepting attitudes: Precursors to or consequences of forced sex. *Violence Against Women*, 7, 876–885.

Maxwell C., Robinson A., Post L. (2003). The nature and predictors of sexual victimization and offending among adolescents. *Journal of Youth and Adolescence*, 32, 465–477.

McMahon, S., Allen, C. T., Postmus, J. L., McMahon, S. M., Peterson, N. A., & Lowe Hoffman, M. (2014). Measuring bystander attitudes and behavior to prevent sexual violence. *Journal of American College Health*, *62*(1): 58-66.

Moore, C. D., & Waterman, C. K. (1999). Predicting self-protection against sexual assault in dating relationships among heterosexual men and women, gay men, lesbians, and bisexuals. *Journal of college student development,* 40:132-140.

Our Watch, VicHealth, & Australia’s National Research Organisation for Women’s Safety (ANROWS). (2015*). Change the story: A shared framework for the primary prevention of violence against women and their children in Australia*. Our Watch.

Porat, R., Gantman, A., Green, S. A., Pezzuto, J. H., & Paluck, E. L. (2024). Preventing sexual violence: A behavioral problem without a behaviorally informed solution. *Psychological science in the public interest*, *25*(1), 4-29.

Taylor N & Mouzos M. (2006). Community attitudes to violence against women survey 2006: A full technical report. Australian Institute of Criminology, Canberra

Taylor, B. G., Stein, N. D., Mumford, E. A., & Woods, D. (2013). Shifting boundaries: An experimental evaluation of a dating violence prevention program in middle schools. *Prevention science*, *14*(1), 64-76.

Tharp, A. T., DeGue, S., Valle, L. A., Brookmeyer, K. A., Massetti, G. M., & Matjasko, J. L. (2013). A systematic qualitative review of risk and protective factors for sexual violence perpetration. *Trauma, Violence, & Abuse*, *14*(2), 133-167.

Thomson, S., Bortoli, L. D., Underwood, C. & Schmid, M. (2019). PISA 2018: Reporting Australia’s Results Volume I Student Performance. Australian Council for Educational Research, Melbourne. https: //research.acer.edu.au/cgi/viewcontent.cgi?article=1035&context=ozpisa.

Valente T. W., Davis R. L. (1999). Accelerating the diffusion of innovations using opinion leaders. *Annals of the American Academy of Political and Social Science*, 566, 55-67.

Weinberger DA, Schwartz GE. (1990). Distress and restraint as superordinate dimensions of adjustment: a typological perspective. *Journal of Personality,* 58(2):381-417.
